# Prevalence and patterns of multimorbidity among linguistic groups of patients receiving home care in Ontario: a retrospective cohort study

**DOI:** 10.1186/s12877-023-04267-5

**Published:** 2023-11-09

**Authors:** Ricardo Batista, Michael Reaume, Rhiannon Roberts, Emily Seale, Emily Rhodes, Ewa Sucha, Michael Pugliese, Claire E. Kendall, Lise M. Bjerre, Louise Bouchard, Denis Prud’homme, Douglas G. Manuel, Peter Tanuseputro

**Affiliations:** 1 Institut du Savoir Montfort, Hôpital Montfort, 202-745A Ch. Montréal Road, Ottawa, ON K1K 0T1 Canada; 2 Ottawa Hospital Research Institute, Ottawa, ON Canada; 3 Department of Internal Medicine, Max Rady College of Medicine, University of Manitoba, Winnipeg, Canada; 4 ICES uOttawa, Ottawa, ON Canada; 5 Bruyère Research Institute, Ottawa, ON Canada; 6 Department of Family Medicine, Faculty of Medicine, University of Ottawa, Ottawa, ON Canada; 7 School of Social and Anthropological Studies, University of Ottawa, Ottawa, ON Canada; 8 Université de Moncton, Nouveau-Brunswick, Canada

**Keywords:** Chronic diseases, Multimorbidity, Home care, Linguistic group, Language barriers, Elders

## Abstract

**Background:**

Prior studies have demonstrated the negative impact of language barriers on access, quality, and safety of healthcare, which can lead to health disparities in linguistic minorities. As the population ages, those with multiple chronic diseases will require increasing levels of home care and long-term services. This study described the levels of multimorbidity among recipients of home care in Ontario, Canada by linguistic group.

**Methods:**

Population-based retrospective cohort of 510,685 adults receiving home care between April 1, 2010, to March 31, 2018, in Ontario, Canada. We estimated and compared prevalence and characteristics of multimorbidity (2 or more chronic diseases) across linguistic groups (Francophones, Anglophones, Allophones). The most common combinations and clustering of chronic diseases were examined. Logistic regression models were used to explore the main predictors of ‘severe’ multimorbidity (defined as the presence of five or more chronic diseases).

**Results:**

The proportion of home care recipients with multimorbidity and severe multimorbidity was 92% and 44%, respectively. The prevalence of multimorbidity was slightly higher among Allophones (93.6%) than among Anglophones (91.8%) and Francophones (92.4%). However, Francophones had higher rates of cardiovascular and respiratory disease (64.9%) when compared to Anglophones (60.2%) and Allophones (61.5%), while Anglophones had higher rates of cancer (34.2%) when compared to Francophones (25.2%) and Allophones (24.3%). Relative to Anglophones, Allophones were more likely to have severe multimorbidity (adjusted OR = 1.04, [95% CI: 1.02–1.06]).

**Conclusions:**

The prevalence of multimorbidity among Ontarians receiving home care services is high; especially for whose primary language is a language other than English or French (i.e., Allophones). Understanding differences in the prevalence and characteristics of multimorbidity across linguistic groups will help tailor healthcare services to the unique needs of patients living in minority linguistic situations.

**Supplementary Information:**

The online version contains supplementary material available at 10.1186/s12877-023-04267-5.

## Introduction

As the population continues to age, the Canadian health care system will be faced with the challenge of providing care to patients with increasing levels of multi morbidity or medical complexity [1]. In 2016, 16.9% of Canadians were over the age of 65; [2] this number is predicted to rise to 25% by 2036 [3]. Older people experience higher rates of health care utilization [4, 5] and are at increased risk of poorer health outcomes [6–10]; this it thought to be a result of the higher prevalence of chronic conditions, multimorbidity, which predisposes these patients to disability and frailty [11–13]. A considerable proportion of Canadians over the age of 65 receive formal support services, including but not limited to home care services. In the fiscal year 2016/17, approximately 760,000 Ontarians (5.8% of the population) received government-funded home care services [14].

Ontario has a publicly funded health care system with a commitment to equitable care for its population, including providing healthcare services in both of Canada’s official languages, English and French [15]. Approximately 5% of Ontarians identify French as their mother tongue; however, the proportion of Francophones can be as high as 30% in regions of the province, such as Eastern Ontario and Northern Ontario [16]. Moreover, approximately 2.5% of Ontarians in 2016 are unable to communicate in one of Canada’s official languages [17]. Despite government efforts to improve the delivery of healthcare services to residents of Ontario living in minority linguistic communities, health disparities related to access, health status (e.g. prevalence of chronic conditions), quality, and safety of care persist across linguistic groups in Ontario [18, 19].

Language is an important socio-cultural factor related to health and wellbeing as well as on access and use of health services [20, 21]. Language barriers impact the amount and quality of information exchanged between a patient and the healthcare providers and their ability to establish rapport, [22] which can impact the quality and safety of care received, and thus affect a patient’s overall health status. While many studies have described the relationship between various socio-demographic characteristics (e.g., sex, age, education, income, immigrant status, and ethnicity) and multimorbidity, [23–25] none have described the prevalence of multimorbidity by linguistic group. In addition, research in Canada have revealed inequalities in the health conditions of official linguistic minorities [19, 26].

The objective of this study was to describe the extent and patterns of multimorbidity among recipients of home care services in Ontario, Canada, stratified by linguistic group. More specifically, we sought to: 1) determine the prevalence of multimorbidity, 2) describe the characteristics and clusters of multimorbidity, and 3) identify the main predictors of severe multimorbidity.

## Methods

### Study design and population

We conducted a population-based retrospective cohort study in Ontario, Canada, using linked administrative databases. Ontario is Canada’s most populous province, and in 2015–2016, 6.7% of households of the province reported that at least one person received formal home care services in the previous 12 months, which is higher than the national average (6.4%) [27]. The province also account for nearly 80% of the clients admitted in home care based on the assessments conducted in 2020–2021 [28]. Reporting of this study follows guidelines for observational studies using routinely collected health care data (see Appendix 1 in Supplemental material).

We included all people in Ontario who received an assessment using the Resident Assessment Instrument for Home Care (RAI-HC) between April 1, 2010, to March 31, 2018. The RAI-HC is a standardized tool to assess individuals' acute and chronic health care needs and is performed for all people who are referred for publicly-funded home healthcare services in Ontario [29]. For patients with more than one RAI assessment, we used the first assessment in the accrual period as the index event (index RAI assessment) to identify individual characteristics. We excluded individuals who were: older than 105 years of age, not eligible for the Ontario Health Insurance Plan (OHIP), and/or did not have a completed language or age variable on their RAI-HC assessment (see Appendix 2 in Supplementary material).

### Data sources

We used administrative databases housed and maintained at ICES (formerly Institute for Clinical Evaluative Sciences). The data were linked using unique encoded identifiers, including: 1) The Resident Assessment Instrument – Home Care (RAI-HC) database, which captures data for Ontario residents receiving publicly funded home care services for at least 60 consecutive days or waiting for admissions to long term care facilities [30]. The RAI-HC database capture data from completed RAI-HC assessments and included baseline sociodemographic (highest-level of education) and health characteristics (Activities of Daily Living Scale [ADL Hierarchy Scale], [31] Cognitive Performance Scale [CPS], [32] Changes in health, End-stage disease, and Signs and Symptoms [CHESS] score, [33] Resource Utilization Grouping III [RUG-III], [34] Charlson index score, a comorbidity index [35] and the number of prescribed medications); 2) the Immigration, Refugees and Citizenship Canada (IRCC) Permanent Resident's Database, which identifies people who immigrated to Canada and became permanent residents after 1985; 3) the Registered Persons Database (RPDB) was used to ascertain demographic characteristics, including age, sex, and resident postal code; 4) the 2016 Statistics Canada Census to generate the study participant’s neighbourhood income quintiles and urban/rural status using postal code data; [36] 5) the Canadian Institute of Health Information (CIHI) Discharge Abstract Database (DAD) and the Ontario Health Insurance Plan (OHIP) database for hospital admissions and physician billings, respectively, to determine chronic disease status using standardized approaches and validated algorithms [37, 38].

In 2007, Ontario’s public healthcare services' planning and distribution were divided into 14 geographically defined local health integration networks (LHINs) [39]. Given the uneven distribution of Francophones in Ontario, we grouped the LHINS into three regions: Northern (comprised of North East and North West LHINs), Eastern (Champlain LHIN), and South-Western (the remaining 11 LHINs).

### Exposure

We identified person’s language using the language variable recorded in RAI-HC from the index RAI assessment. During RAI-HC assessments, language is ascertained by the interviewer and, if necessary, by asking the home care recipient report their primary language [40]. We classified Anglophones and Francophones as individuals that spoke English and French, respectively. All remaining individuals were considered as Allophones. We excluded individuals who communicate with non-spoken languages (e.g., artificial languages, sign languages).

### Outcomes

Our main outcomes were a) the prevalence of individual chronic disease(s) (see Appendix 2 in Supplementary material) and b) the prevalence of multimorbidity (defined as two or more chronic diseases). The prevalence of chronic diseases was ascertained using algorithms validated and applied in previous studies [38, 41]. We categorized individuals based on their number of chronic diseases (0, 1, 2, 3, 4 and 5 +), and two levels of multimorbidity, 2 + and 3 + [42, 43]. Home care recipients with higher numbers of conditions have been shown to have greater functional decline and poorer health-related quality of life; [41, 44–46] we used the category of five or more chronic diseases (5 + comorbidities) to assess the risk of higher levels of comorbidity by language group in our study.

We used two approaches to examine the patterns of multimorbidity. First, we derived the five most common co-occurring clusters of conditions within each level of comorbidity (i.e., 2, 3, 4, 5) and measured their prevalence (number of individuals with the most common combination in each level of multimorbidity divided by the number of individuals in this level). Second, we followed a non-data driven approach to group the prevalent chronic conditions that uses a clinical criteria, such as the risk adjustment model of the Medicare and Medicaid Services – Hierarchical Conditions Categories (CMS/HCC) [47]. This model applies a risk assessment criteria to create diagnostic categories that are clinically meaningful to clinicians, due to its interpretability and utility for disease management and quality monitoring [48–50]. Similar approaches have been used to define disease groups to study health outcomes and quality of life, and to provide patient-centred care to individuals with multimorbidity [50–52]. Therefore, seven predefined clinical clusters were created (Table 1). First, we included multimorbid individuals with cancer, dementia, or stroke-related condition into one individual cluster. Then, we grouped the remaining multimorbid individuals into the cluster of their first diagnosis.

**Table 1:** Clinical clusters of chronic diseases

Functional Cluster	Chronic disease
C1. Cancer	All Cancers
C2. Cardio-Respiratory	AMI, Arrhythmia, Asthma, CHF, Coronary Heart Disease, Hypertension, COPD
C3. Mental disorders	Mood, anxiety, depression and other nonpsychotic disorders, Other mental health conditions
C4. Metabolic-GI-Renal	Diabetes, IBD, Renal disease
C5. Muscle-skeletal	Osteoarthritis, Rheumatoid arthritis, Other Arthritis (Synovitis, Fibrositis, Connective tissue disorders, Ankylosing spondylitis, Gout Traumatic arthritis, pyogenic arthritis, Joint derangement, Dupuytren's contracture, Other MSK disorders), Osteoporosis
C6. Dementia	Dementia
C7. Stroke	Stroke (excluding transient ischemic attack)

*AMI* acute myocardial infarction, *CHF* congestive health failure, *COPD* chronic obstructive pulmonary disease, *IBD* inflammatory bowel disease, *RA* rheumatoid arthritis, *MSK* Muscle-skeletal

### Analysis

We used frequency measures to compare the cohort’s baseline sociodemographic and health characteristics by linguistic group (Francophones, Anglophones, Allophones). We used descriptive analyses to compare the prevalence and characteristics of the comorbidities across linguistic groups.

We fitted multivariable logistic regression models to explore the main predictors of ‘severe’ multimorbidity, adjusted for patient age group (ref. < 50), sex (ref. male), immigration status (ref. long-term residents), neighbourhood income quintile (ref. Q5), rurality (ref. urban), region of the province (ref. southwestern) and the functional health variables (ADL, CPS, CHESS [ref. the lowest scores]).

### Ethics approval

ICES is a prescribed entity under Sect. 45 of Ontario's Personal Health Information Protection Act. Section 45 authorizes ICES to collect personal health information, without consent, for the purpose of analysis or compiling statistical information with respect to the management of, evaluation or monitoring of, allocation of resources to or planning for all or part of the health system. Projects that use data collected by ICES under Sect. 45 of PHIPA, and use no other data, are exempt from REB review. The use of the data in this project is authorized under Sect. 45 and approved by ICES’ Privacy and Legal Office.

## Results

A total of 510,685 adults receiving home care services in Ontario met the eligibility criteria and were included in this study. Table 2 presents the cohort’s sociodemographic characteristics. The majority of the cohort were Anglophones (80.2%), followed by Allophones (17.5%) and Francophones (2.3%), 65 years or older (78.9%), and women (58.8%).

**Table 2:** Sociodemographic characteristics by linguistic group

**Characteristics**		**Anglophone**		**Francophone**		**Allophone**		***Total***	
		*(N* = *409,578)*		*(N* = *11,907)*		*(N* = *89,200)*		*(N* = *510,685)*	
		**#**	**%**	**#**	**%**	**#**	**%**	**#**	**%**
**Sex**	Female	238,950	58.3	7,250	60.9	54,006	60.5	*300,206*	*58.8*
	Male	170,628	41.7	4,657	39.1	35,194	39.5	*210,479*	*41.2*
**Age group**	< 50	29,004	7.1	484	4.1	2,508	2.8	*31,996*	*6.3*
	50–64	67,277	16.4	1,567	13.2	6,798	7.6	*75,642*	*14.8*
	65 +	313,297	76.5	9,856	82.8	79,894	89.6	*403,047*	*78.9*
**Immigration**	Long-term Resident	406,177	99.2	11,828	99.3	80,171	89.9	*498,176*	*97.6*
**status**	Immigrant	3,401	0.8	79	0.7	9,029	10.1	*12,509*	*2.4*
**Highest level of**	Less than High school	83,484	20.4	4,927	41.4	32,047	35.9	*120,458*	*23.6*
**education**	High school completed	68,001	16.6	1,447	12.2	7,077	7.9	*76,525*	*15.0*
	Some post-secondary	57,775	14.1	1,295	10.9	6,542	7.3	*65,612*	*12.8*
	Univ./Post-sec completed	43,457	10.6	895	7.5	5,616	6.3	*49,968*	*9.8*
**Neighborhood**	Q1 (Lowest)	101,561	24.8	3,350	28.1	24,610	27.6	*129,521*	*25.4*
**income**	Q2	88,490	21.6	2,867	24.1	21,384	24.0	*112,741*	*22.1*
**quintile**	Q3	77,615	18.9	2,355	19.8	17,541	19.7	*97,511*	*19.1*
	Q4	71,583	17.5	1,877	15.8	14,659	16.4	*88,119*	*17.3*
	Q5 (Highest)	69,246	16.9	1,421	11.9	10,791	12.1	*81,458*	*16.0*
**Area of**	Urban	348,928	85.2	8,411	70.6	87,527	98.1	*444,866*	*87.1*
**residence**	Rural	60,329	14.7	3,477	29.2	1,614	1.8	*65,420*	*12.8*
**Region of**	Eastern	28,448	6.9	5,589	46.9	3,485	3.9	*37,522*	*7.3*
**residence**^a^	Northern	31,637	7.7	4,380	36.8	2,430	2.7	*38,447*	*7.5*
	Southwest	349,492	85.3	1,938	16.3	83,285	93.4	*434,715*	*85.1*

^a^Regions: Northern (Northeast and Northwest LHINs), Eastern (Champlain LHIN), Southwest (remaining 11 LHINs). Ontario is organized in 14 health regions or LHINs (Local Health Integration Network)

A larger proportion of Francophones lived in rural areas (29.2%) compared to Anglophones (14.7%) and Allophones (1.8%). Francophones made a larger proportion of the population in the Northern (36.8%) and Eastern (46.9%) regions of the province. Overall, Anglophones were more highly educated (10.6% had completed a university degree), compared to Francophones (7.5%) and Allophones (6.3%). Anglophones were also more likely to live in the highest income quintile neighbourhood, with nearly 35% of Anglophones living in neighbourhoods with a household income within the 4th or 5th quintiles of income, compared to less than 30% of Allophones and Francophones. Allophones had worse physical and cognitive performance compared to Anglophones and Francophones (see the health-related characteristics of the cohort in Table S1 of Supplementary material).

### Chronic diseases and multimorbidity

Overall, 92% of the cohort had two or more chronic diseases, and 44% had 5 or more diseases (Table 3). Compared to Anglophones and Francophones, Allophones had the highest proportion of chronic conditions across all categories of comorbidities (2 + , 3 + , 4 + , 5 +).

**Table 3:** Prevalence of chronic diseases by linguistic group—(*N* = 510,685)

**Health conditions**	**Anglophone** *(N* = *409,578)*		**Francophone** *(N* = *11,907)*		**Allophone** *(N* = *89,200)*		**Total** *(N* = *510,685)*	
	#	%	#	%	#	%	#	%
**Level of multimorbidity**								
2 + diseases	375,849	91.8	11,003	92.4	83,527	93.6	*470,379*	*92.1*
3 + diseases	325,253	79.4	9,636	80.9	74,011	83.0	*408,900*	*80.1*
4 + diseases	254,230	62.1	7,650	64.2	58,565	65.7	*320,445*	*62.7*
5 + diseases	178,007	43.5	5,426	45.6	41,248	46.2	*224,681*	*44.0*
Charlson index score (mean-SD)	1.62	2.04	1.64	1.96	1.56	1.91	*1.63*	*1.87*
**Prevalence of chronic diseases**								
AMI	6,822	1.7	226	1.9	1,370	1.5	*8,418*	*1.6*
Arrhythmia	87,225	21.3	2,350	19.7	20,564	23.1	*110,139*	*21.6*
Asthma	74,941	18.3	2,352	19.8	16,694	18.7	*93,987*	*18.4*
Cancer	134,630	32.9	2,854	24.0	21,011	23.6	*158,495*	*31.0*
Congestive Heart Failure (CHF)	92,830	22.7	3,052	25.6	22,301	25.0	*118,183*	*23.1*
COPD	82,749	20.2	3,258	27.4	12,227	13.7	*98,234*	*19.2*
Coronary Heart Disease (CHD)	142,916	34.9	4,617	38.8	32,825	36.8	*180,358*	*35.3*
Dementia	64,013	15.6	2,185	18.4	17,035	19.1	*83,233*	*16.3*
Diabetes	142,894	34.9	4,624	38.8	40,408	45.3	*187,926*	*36.8*
Hypertension	306,181	74.8	9,201	77.3	74,222	83.2	*389,604*	*76.3*
Inflammatory Bowel Disease (IBD)	5,553	1.4	113	1.0	461	0.5	*6,127*	*1.2*
Other mental health conditions	39,790	9.7	1,060	8.9	6,843	7.7	*47,693*	*9.3*
Non-psych. mood & anxiety dis	89,229	21.8	2,634	22.1	17,030	19.1	*108,893*	*21.3*
Osteoarthritis	290,286	70.9	8,502	71.4	65,248	73.2	*364,036*	*71.3*
Osteoporosis	55,145	13.5	1,433	12.0	15,794	17.7	*72,372*	*14.2*
Renal disease	67,734	16.5	1,950	16.4	15,571	17.5	*85,255*	*16.7*
Rheumatoid arthritis	16,386	4.0	497	4.2	2,830	3.2	*19,713*	*3.9*
Stroke	55,090	13.5	1,502	12.6	13,215	14.8	*69,807*	*13.7*

*AMI* acute myocardial infarction, *CHF* congestive health failure, *COPD* chronic obstructive pulmonary disease, *IBD* inflammatory bowel disease, *RA* rheumatoid arthritis

The most prevalent chronic diseases were hypertension (76.3%), osteoarthritis [OA] (71.3%), diabetes (36.8%) and coronary heart diseases [CHD] (35.3%). Allophones had the highest prevalence of hypertension (83.2%), OA (73.2%), diabetes (45.3%), dementia (19.1%), osteoporosis (17.7%) and stroke (14.8%). Francophones had the highest prevalence of major cardiovascular diseases such as CHD (38.8%), congestive heart failure [CHF] (25.6%), acute myocardial infarction [AMI] (1.9%), as well as COPD (27.4%). The prevalence of cancer was significantly higher among Anglophones (32.9%) than among Francophones (24.0%) and Allophones (23.6%).

### Patterns of multimorbidity clusters

There were no significant differences in the patterns of the top-five common disease combinations per multimorbidity level across linguistic groups (see Table S2 in Supplementary material). The order of the disease combinations was similar across the three linguistic groups, except for the category of 5 or more diseases for allophones and francophones, in which the combination including cancer was the second most common, whereas it was the first one for anglophones.

#### Top-five combinations of chronic diseases

Hypertension and OA are the most frequent diseases, with at least one appearing in all the top-five common combinations (see Table S2 in Supplementary material). Excluding these two chronic diseases, major cardiovascular diseases (e.g., CHD, CHF), dementia, diabetes, and cancer were the most frequent diseases in the top-five combinations. According to the *type of disease* present in a combination, Anglophones were overrepresented in combinations categories with cancer, whereas allophones overrepresented in combinations with hypertension, OA, diabetes, and CHD. Francophones were overrepresented in combinations with dementia.

#### Clinical clusters of diseases

**Fig. 1:**
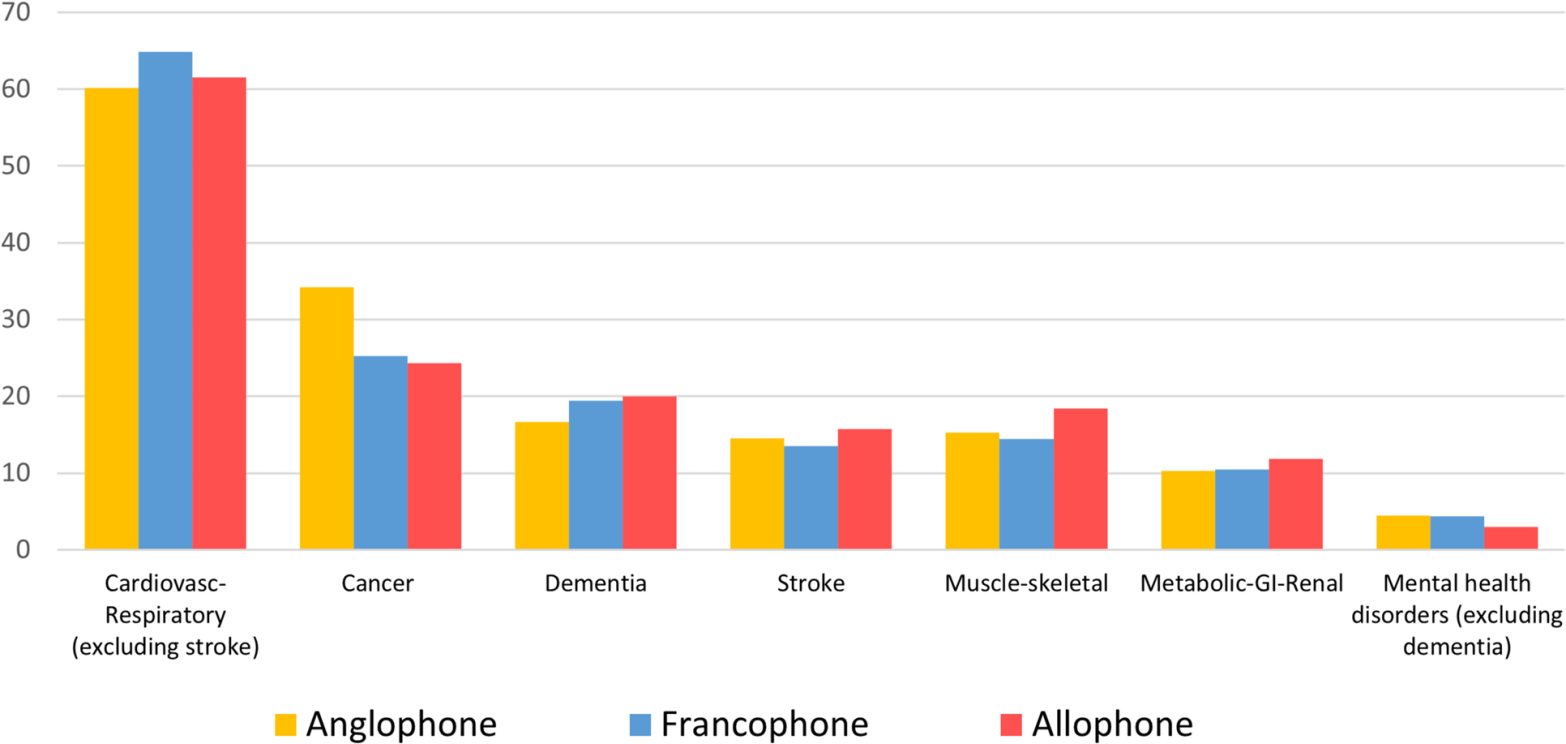
The proportion of clinical clusters by linguistic group

Cardiovascular and respiratory diseases (60.6%) and Cancer (32.2%) clusters were the most important functional clusters of chronic diseases (Fig. 1). These two clusters were the most prevalent across all linguistic groups.

The proportion of Francophones was significantly higher in the clusters of cardiovascular-respiratory diseases (64.9%) compared to Anglophones (60.2%) and Allophones (61.5%) (p < 0.001). Consistent with the prevalence of individual diseases, the cancer cluster was significantly more prominent among Anglophones (34.2%) than Francophones (25.2%) and Allophones (24.3%) (p < 0.001). Similarly, a significantly higher proportion of Allophones fell in the dementia cluster than the other linguistic groups. Allophones were notably overrepresented in the stroke and muscle-skeletal diseases clusters (Fig. 1).

## Predictors of severe multimorbidity

**Fig. 2:**
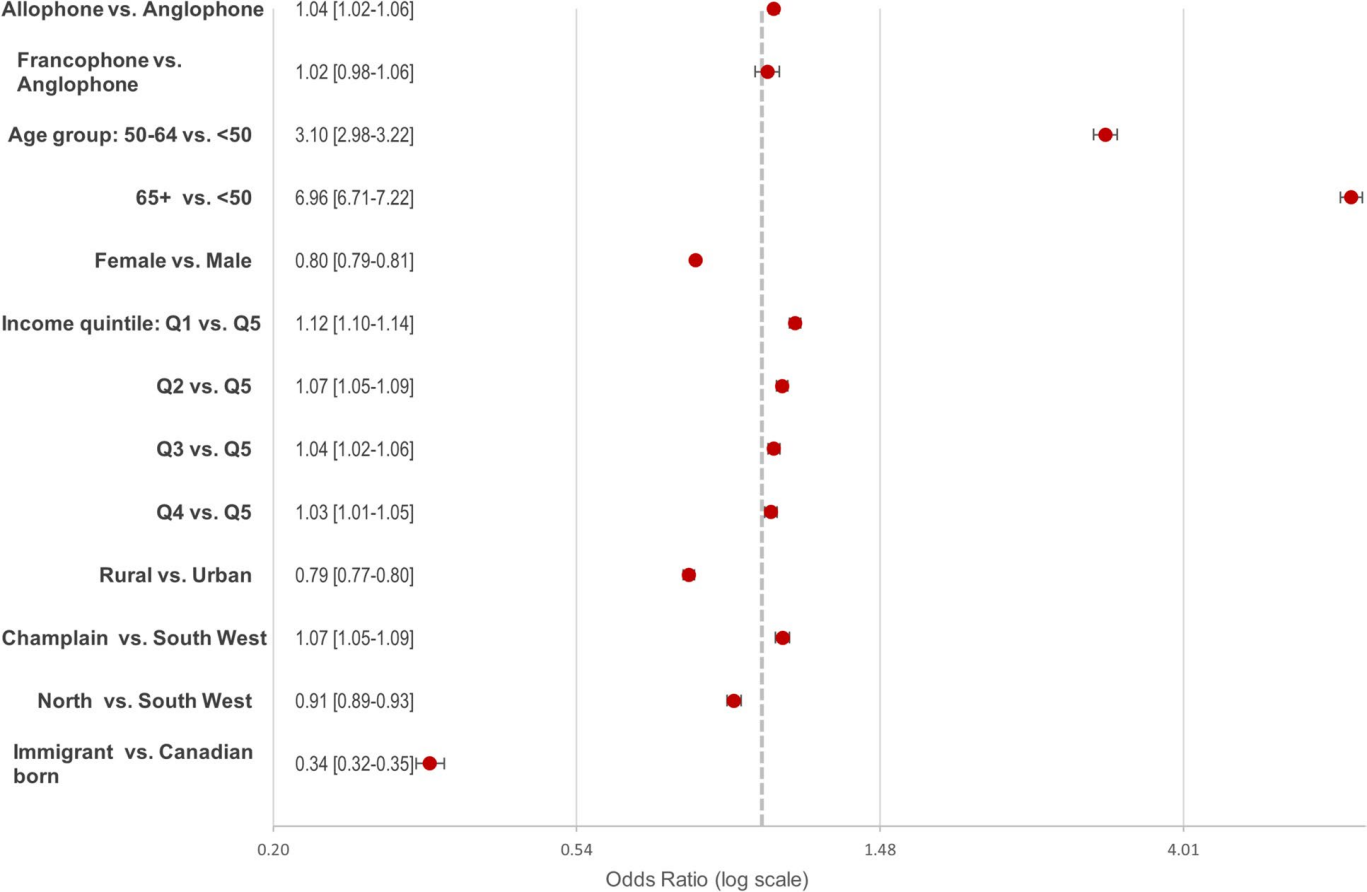
Multivariable analysis of the association between sociodemographic and health characteristics and severe multimorbidity (5 or more chronic diseases) (adjusted OR*, 95% CI). * Multivariable logistic regression model, adjusted by age, sex, neighborhood income level, immigrant status, rurality, area, and health characteristics

The multivariable regression analysis showed that, relative to Anglophones, Allophones had significantly greater odds of having five or more chronic diseases (adjusted OR = 1.04, [95% CI: 1.02–1.06]) (Fig. 2). Compared to those under 50 years of age, patients in age groups 50–64 years and 65 years and over were three (aOR = 3.10, [95% CI: 2.98–3.22]) and seven (aOR = 6.96, [95% CI: 6.71–7.22]) times more likely to have severe multimorbidity, respectively. Living in Eastern Ontario was also associated with higher odds of severe multimorbidity (aOR = 1.07, [95% CI: 1.05–1.09]). Conversely, being female (aOR = 0.80, [95% CI: 0.79–0.81]), immigrant (aOR = 0.34, [95% CI: 0.32–0.35]) and residing in rural areas (aOR = 0.79, [95% CI: 0.77–0.80]) were significantly associated with lower odds of having severe multimorbidity.

There was also a clear gradient between neighbourhood income level and severe multimorbidity, from wealthier quintile 4 (aOR = 1.03, [95% CI: 1.01–1.05]) to the poorest quintile (aOR = 1.12, [95% CI: 1.10–1.14]), relative to the wealthiest Q5.

## Discussion

This study described multimorbidity in Ontario residents receiving home care, stratified by linguistic group. As expected, a high proportion (92%) of individuals receiving home care have multimorbidity (defined as two or more chronic diseases). The high prevalence is consistent with other studies which found higher levels of multimorbidity among older people and receiving long-term care [53, 54]. Across linguistic groups, Allophones had the highest proportion of multimorbidity, followed by Francophones, then Anglophones.

When considering multimorbidity by combinations of chronic diseases, we found that Hypertension or OA appeared in all top-5 combinations, a finding consistent with previous population-based studies in Ontario [38]. Excluding these two chronic diseases, we found that major cardiovascular diseases (i.e. CHD and CHF), dementia, diabetes, and cancer were the most frequent diseases appearing in common combinations. This pattern was consistent across the linguistic groups. Also, the combinations of chronic diseases showed no differences by linguistic characteristics.

We then examined clusters of chronic diseases, which may be more clinically relevant for healthcare providers. Overall, the most prevalent clinical clusters were cardiovascular/respiratory disease (excluding stroke) and dementia, a finding that is consistent with prior studies of populations of older recipients of home care services [37]. Francophones were overrepresented in the cardiovascular/respiratory disease, which is likely due to the higher rates of cardiovascular risk factors (e.g., smoking, dyslipidemia, family history of CVD), which has been reported in previous studies [55, 56]. Furthermore, a report on the health of seniors in Ontario showed that Franco-Ontarians had higher rates of obesity, especially those living in minority linguistic communities [57]. The overrepresentation of Anglophones in the cancer cluster could be related to a higher cancer survival levels observed in the southwest region of the province, which is predominantly anglophone [58]. This may be also related to health seeking behaviours, as anglophones with cancer seek out home care more often and earlier in life, whereas language and cultural barriers can influence cancer care seeking behaviours among non-anglophone patients [59, 60].

Previous studies have reported an association between high levels of multimorbidity with disability, frailty and poor health outcomes [61–63]. We hypothesized that linguistic minorities (e.g., Francophones and Allophones in Ontario) would have higher levels of multimorbidity due to the effect of language barriers to health services. We found that Allophones were significantly more likely to have 5 + multimorbidity. This may be due to the fact that Allophones may not seek health services due to their limited capacity to communicate in Canada’s official languages or may not seek out publicly funded home care services due to cultural preferences, differences in informal health care services (e.g., large households, family structures), leading to more health complications and worse health outcomes. Moreover, a large proportion of Allophones are recent immigrants, who face additional barriers accessing and using health services [64, 65]. As this linguistic group has poor ability to communicate or are not proficient in one of Canada’s official languages, it makes them more susceptible to barriers in accessing health care services and receiving appropriate care, leading to poorer health outcomes. However, the independent protective effect of immigrants might be related to the healthy immigrant effect, which is widely documented, [66] as well as their lower levels of health care seeking behaviours that have been identified among immigrants [67]. Unfortunately, we were not able to account for other migration-related factors (e.g. length of stay in Canada) to assess that assumption in this study.

## Strengths and limitations

This study has several strengths, notably its use of a large population-based cohort and validated datasets. However, this study also has limitations. We obtained an individual's primary language from the RAI-HC assessments. During these assessments, interviewers are instructed to determine the home care recipient's primary language by listening and observing and, if necessary, by asking the home care recipient to specify his or her primary language. For Ontarians who speak multiple languages, it is unclear how interviewers assign primary language. Also, the interviewers do not assess language proficiency, which is particularly important for Ontarians that speak multiple languages. However, our group's preliminary analyses have shown that the language variable of the RAI-HC database have a high agreement (kappa = 0.76) with self-reported language at home from the Canadian Community Health Survey. (Batista et al., unpublished data, 2019). Finally, the approach used to create the clusters could be a limitation, as it can affect the generalizability of the findings and comparisons with other data-driven approaches for clustering chronic diseases.

## Conclusions and implications

The prevalence of multimorbidity among Ontarians receiving home care services is high. There exist important clinical differences in the prevalence and characteristics of disease burden across linguistic groups. Understanding the interaction between language and multimorbidity will allow policy makers to implement patient-oriented healthcare strategies that address the needs of linguistic minorities, who face barriers to accessing appropriate healthcare services. This study found that home care recipients whose primary language was other than English or French were more likely to have severe multimorbidity. Hence, healthcare systems should identify individuals living in minority linguistic situations and implement strategies (e.g. care coordinators which can facilitate access to home care services, interpretation services, social work) to provide care to patients with important communication barriers and also more complex care needs. However, additional studies are needed to understand the effect of other linguistic factors (e.g., language of service, patient-provider language discordance, use of interpreters) on the healthcare and the health outcomes of people living with multimorbidity in Ontario.

### Supplementary Information

**Additional file 1:** **Appendix 1.** The RECORD statement – checklist of items, extended from the STROBE statement. **Appendix 2.** Cohort creation flowchart. **Appendix 3.** List of diagnosis codes for defining the 18 selected chronic conditions and Clinical clusters. **Appendix 4.** Supplementary tables. **Table S1.** Clinical characteristics of the cohort by linguistic group - (*N*=510,685). **Table S2.** Top-five combinations of diseases across multimorbidity levels (2-5 diseases), by linguistic group (*N*=470,379). **Table S3.** Risk of severe multimorbidity (5 or more chronic diseases) and linguistic characteristics (adjusted OR*, 95% CI).

## Data Availability

The data set from this study is held securely in coded form at ICES. While data sharing agreements prohibit ICES from making the data set publicly available, access may be granted to those who meet pre-specified criteria for confidential access, available at www.ices.on.ca/DAS. The full data set creation plan and underlying analytic code are available from the authors upon request, understanding that the computer programs may rely upon coding templates or macros that are unique to ICES and are therefore either inaccessible or may require modification.

## Abbreviations

ADL: Activities of Daily Living
AMI: Acute myocardial infarction
AMI: Acute myocardial infarction
CHD: Coronary heart diseases
CHESS: Changes in health, End-stage disease, and Signs and Symptoms
CHF: Congestive health failure
CHF: Congestive heart failure
CIHI: Canadian Institute of Health Information
COPD: Chronic obstructive pulmonary disease,
CPS: Cognitive Performance Scale
DAD: Discharge Abstract Database
IBD: Inflammatory bowel disease
ICES: Formerly, Institute for Clinical Evaluative Sciences
IRCC: Immigration, Refugees and Citizenship Canada
LHINs: Local health integration networks
MSK: Muscle-skeletal
OA: Osteoarthritis
OHIP: Ontario Health Insurance Plan
RA: Rheumatoid arthritis
RAI-HC: Resident Assessment Instrument for Home Care
RPDB: Registered Persons Database
RUG-III: Resource Utilization Grouping III
