# Supplementary material for: Prevalence and patterns of multimorbidity among linguistic groups of patients receiving home care in Ontario: a retrospective cohort study
Source: BMC Geriatr. 2023 Nov 9;23:725. doi: 10.1186/s12877-023-04267-5 (PMC10634019; doi:10.1186/s12877-023-04267-5)
Supplement: Supplementary file 1 — Additional file 1: Appendix 1. The RECORD statement – checklist of items, extended from the STROBE statement. Appendix 2. Cohort creation flowchart. Appendix 3. List of diagnosis codes for defining the 18 selected chronic conditions and Clinical clusters. Appendix 4. Supplementary tables. Table S1. Clinical characteristics of the cohort by linguistic group - (N=510,685). Table S2. Top-five combinations of diseases across multimorbidity levels (2-5 diseases), by linguistic group (N=470,379). Table S3. Risk of severe multimorbidity (5 or more chronic diseases) and linguistic characteristics (adjusted OR*, 95% CI). [file 12877_2023_4267_MOESM1_ESM.docx]

**Prevalence and patterns of multimorbidity among linguistic groups of patients receiving home care in Ontario: a retrospective cohort study**

Supplementary material

Appendix 1. The RECORD statement – checklist of items, extended from the STROBE statement.

Appendix 2. Cohort creation flowchart

Appendix 3. List of diagnosis codes for defining the 18 selected chronic conditions and Clinical clusters

Appendix 4. Supplementary tables

# Supplementary data

**Appendix 1. The RECORD statement – checklist of items, extended from the STROBE statement.**

|  | **Item No.** | **STROBE items** | **Location in manuscript where items are reported** | **RECORD items** | **Location in manuscript where items are reported** |
| --- | --- | --- | --- | --- | --- |
|  | 1 | (a) Indicate the study’s design with a commonly used term in the title or the abstract (b) Provide in the abstract an informative and balanced summary of what was done and what was found |  | RECORD 1.1: The type of data used should be specified in the title or abstract. When possible, the name of the databases used should be included.  RECORD 1.2: If applicable, the geographic region and timeframe within which the study took place should be reported in the title or abstract.  RECORD 1.3: If linkage between databases was conducted for the study, this should be clearly stated in the title or abstract. | Abstract  Abstract  Abstract |
| Background rationale | 2 | Explain the scientific background and rationale for the investigation being reported |  |  | Introduction |
| Objectives | 3 | State specific objectives, including any prespecified hypotheses |  |  | Introduction |
| Study Design | 4 | Present key elements of study design early in the paper |  |  | Methods |
| Setting | 5 | Describe the setting, locations, and relevant dates, including periods of recruitment, exposure, follow-up, and data collection |  |  | Methods |
| Participants | 6 | *(a) Cohort study* - Give the eligibility criteria, and the sources and methods of selection of participants. Describe methods of follow-up  *Case-control study* - Give the eligibility criteria, and the sources and methods of case ascertainment and control selection. Give the rationale for the choice of cases and controls  *Cross-sectional study* - Give the eligibility criteria, and the sources and methods of selection of participants  *(b) Cohort study* - For matched studies, give matching criteria and number of exposed and unexposed  *Case-control study* - For matched studies, give matching criteria and the number of controls per case |  | RECORD 6.1: The methods of study population selection (such as codes or algorithms used to identify subjects) should be listed in detail. If this is not possible, an explanation should be provided.  RECORD 6.2: Any validation studies of the codes or algorithms used to select the population should be referenced. If validation was conducted for this study and not published elsewhere, detailed methods and results should be provided.  RECORD 6.3: If the study involved linkage of databases, consider use of a flow diagram or other graphical display to demonstrate the data linkage process, including the number of individuals with linked data at each stage. | Methods  Methods  Methods |
| Variables | 7 | Clearly define all outcomes, exposures, predictors, potential confounders, and effect modifiers. Give diagnostic criteria, if applicable. |  | RECORD 7.1: A complete list of codes and algorithms used to classify exposures, outcomes, confounders, and effect modifiers should be provided. If these cannot be reported, an explanation should be provided. | Methods, Supplemental information |
| Data sources/ measurement | 8 | For each variable of interest, give sources of data and details of methods of assessment (measurement).  Describe comparability of assessment methods if there is more than one group |  |  | Methods |
| Bias | 9 | Describe any efforts to address potential sources of bias |  |  | Limitations |
| Study size | 10 | Explain how the study size was arrived at |  |  | Methods |
| Quantitative variables | 11 | Explain how quantitative variables were handled in the analyses. If applicable, describe which groupings were chosen, and why |  |  | Methods |
| Statistical methods | 12 | (a) Describe all statistical methods, including those used to control for confounding  (b) Describe any methods used to examine subgroups and interactions  (c) Explain how missing data were addressed  (d) *Cohort study* - If applicable, explain how loss to follow-up was addressed  *Case-control study* - If applicable, explain how matching of cases and controls was addressed  *Cross-sectional study* - If applicable, describe analytical methods taking account of sampling strategy  (e) Describe any sensitivity analyses |  |  | Methods |
| Data access and cleaning methods |  | .. |  | RECORD 12.1: Authors should describe the extent to which the investigators had access to the database population used to create the study population.  RECORD 12.2: Authors should provide information on the data cleaning methods used in the study. | Methods |
| Linkage |  | .. |  | RECORD 12.3: State whether the study included person-level, institutional-level, or other data linkage across two or more databases. The methods of linkage and methods of linkage quality evaluation should be provided. | Methods |
| Participants | 13 | (a) Report the numbers of individuals at each stage of the study (*e.g.*, numbers potentially eligible, examined for eligibility, confirmed eligible, included in the study, completing follow-up, and analysed)  (b) Give reasons for non-participation at each stage.  (c) Consider use of a flow diagram |  | RECORD 13.1: Describe in detail the selection of the persons included in the study (*i.e.,* study population selection) including filtering based on data quality, data availability and linkage. The selection of included persons can be described in the text and/or by means of the study flow diagram. | Methods |
| Descriptive data | 14 | (a) Give characteristics of study participants (*e.g.*, demographic, clinical, social) and information on exposures and potential confounders  (b) Indicate the number of participants with missing data for each variable of interest  (c) *Cohort study* - summarise follow-up time (*e.g.*, average and total amount) |  |  | Results, Table 1 and Table S1 in Supplemental information |
| Outcome data | 15 | *Cohort study* - Report numbers of outcome events or summary measures over time  *Case-control study* - Report numbers in each exposure category, or summary measures of exposure  *Cross-sectional study* - Report numbers of outcome events or summary measures |  |  | Results, Table 2, Table S2 in Supplemental information |
| Main results | 16 | (a) Give unadjusted estimates and, if applicable, confounder-adjusted estimates and their precision (e.g., 95% confidence interval). Make clear which confounders were adjusted for and why they were included  (b) Report category boundaries when continuous variables were categorized  (c) If relevant, consider translating estimates of relative risk into absolute risk for a meaningful time period |  |  | Results, Figures 1 and 2 |
| Other analyses | 17 | Report other analyses done—e.g., analyses of subgroups and interactions, and sensitivity analyses |  |  | N/A |
| Key results | 18 | Summarise key results with reference to study objectives |  |  | Discussion |
| Limitations | 19 | Discuss limitations of the study, taking into account sources of potential bias or imprecision. Discuss both direction and magnitude of any potential bias |  | RECORD 19.1: Discuss the implications of using data that were not created or collected to answer the specific research question(s). Include discussion of misclassification bias, unmeasured confounding, missing data, and changing eligibility over time, as they pertain to the study being reported. | Limitations |
| Interpretation | 20 | Give a cautious overall interpretation of results considering objectives, limitations, multiplicity of analyses, results from similar studies, and other relevant evidence |  |  | Discussion, Conclusions |
| Generalisability | 21 | Discuss the generalisability (external validity) of the study results |  |  | Limitations |
| Funding | 22 | Give the source of funding and the role of the funders for the present study and, if applicable, for the original study on which the present article is based |  |  | Acknowledgement and Funding |
| Accessibility of protocol, raw data, and programming code |  | .. |  | RECORD 22.1: Authors should provide information on how to access any supplemental information such as the study protocol, raw data, or programming code. |  |

*Reference: Benchimol EI, Smeeth L, Guttmann A, Harron K, Moher D, Petersen I, Sørensen HT, von Elm E, Langan SM, the RECORD Working Committee. The REporting of studies Conducted using Observational Routinely-collected health Data (RECORD) Statement. *PLoS Medicine* 2015; in press.

*Checklist is protected under Creative Commons Attribution ([CC BY](http://creativecommons.org/licenses/by/4.0/)) license.

**Appendix 2. Cohort creation flowchart**

**
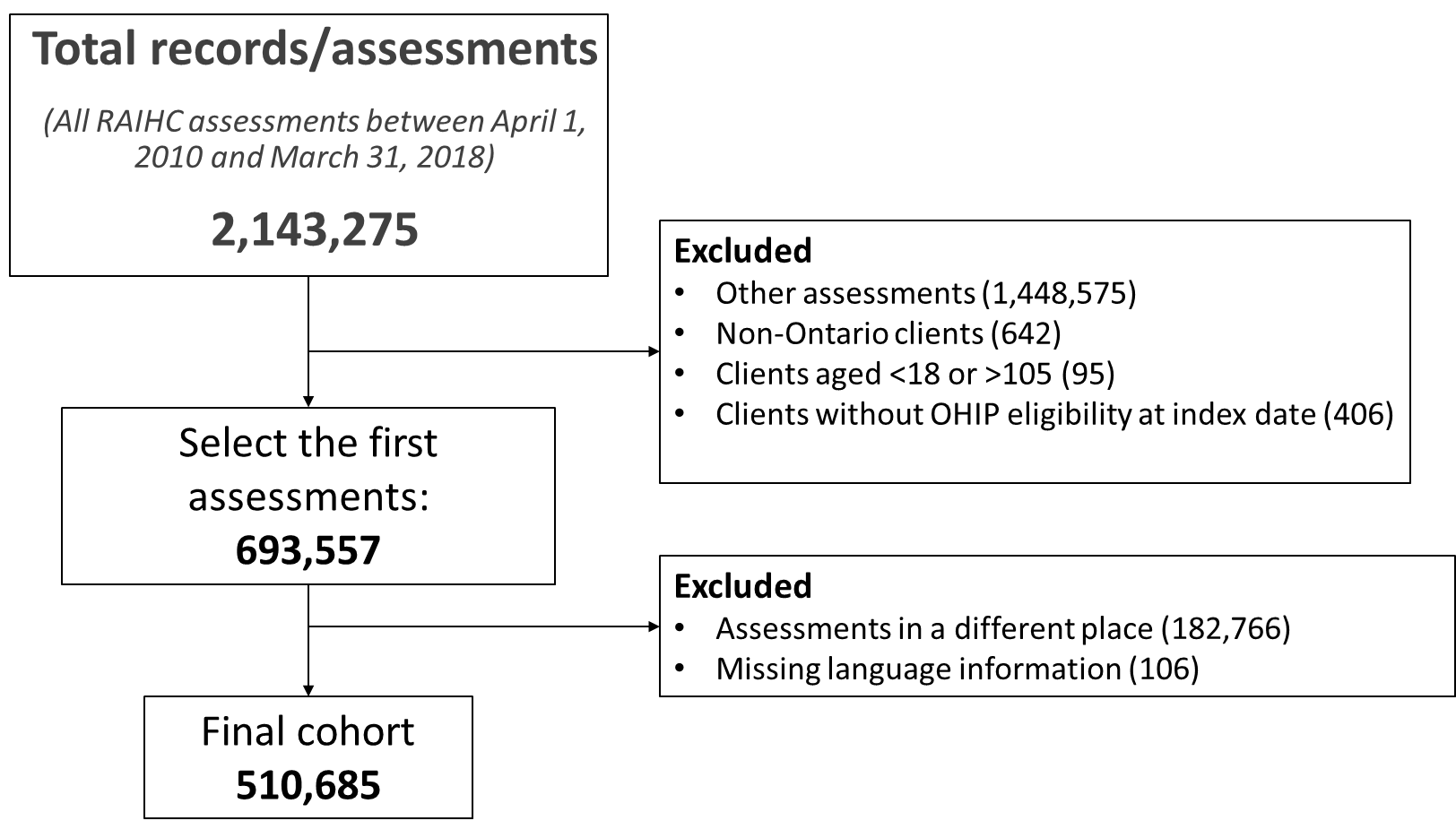
**

**Appendix 3. List of diagnosis codes for defining the 18 selected chronic conditions and Clinical clusters**

| **Clinical Cluster** | **Chronic conditions** | **ICD 9 / OHIP** | **ICD 10** |
| --- | --- | --- | --- |
| Cancer | Cancer (all) | 140-239 | C00-C26, C30-C44, C45-C97 |
| Cardio- | AMI | 410 | I21, I22 |
| Respiratory | Arrhythmia | 427.3 (DAD) / 427 (OHIP) | I48.0, I48.1 |
|  | Asthma | 493 | J45 |
|  | CHF | 428 | I500, I501, I509 |
|  | COPD | 491, 492, 496 | J41, J43, J44 |
|  | Coronary Heart Disease | 411-414 | I20, I22-I25 |
|  | Hypertension | 401, 402, 403, 404, 405 | I10, I11, I12, I13, I15 |
|  | Stroke (exclude transient ischemic attack) | 430, 431, 432, 434, 436 | I60-I64 |
| Mental disorders | Dementia | 290, 331 (OHIP) / 046.1, 290.0, 290.1, 290.2, 290.3, 290.4, 294, 331.0, 331.1, 331.5, F331.82 (DAD) | F00, F01, F02, F03, G30 |
|  | Mood, anxiety, depression and other nonpsychotic disorders | 296, 300, 309, 311 | F30, F31, F32, F33, F34 (excl. F34.0), F38, F39, F40, F41, F42, F43.1, F43.2, F43.8, F44, F45.0, F45.1, F45.2, F48, F53.0, F68.0, F93.0, F99 |
|  | Other mental health conditions | 291, 292, 295, 297, 298, 299, 301, 302, 303, 304, 305, 306, 307, 313, 314, 315, 319 | F04, F050, F058, F059, F060, F061, F062, F063, F064, F07, F08, F10, F11, F12, F13, F14, F15, F16, F17, F18, F19, F20, F21, F22, F23, F24, F25, F26, F27, F28, F29, F340, F35, F36, F37, F430, F439, F453, F454, F458, F46, F47, F49, F50, F51, F52, F531, F538, F539, F54, F55, F56, F57, F58, F59, F60, F61, F62, F63, F64, F65, F66, F67, F681, F688, F69, F70, F71, F72, F73, F74, F75, F76, F77, F78, F79, F80, F81, F82, F83, F84, F85, F86, F87, F88, F89, F90, F91, F92, F931, F932, F933, F938, F939, F94, F95, F96, F97, F98 |
| Metabolic- | Diabetes | 250 | E08 - E13 |
| GI-Renal | IBD |  | K58 |
|  | Renal disease | 403, 404, 584, 585, 586, v451 | N17, N18, N19, T82.4, Z49.2, Z99.2 |
| Muscle- | Osteoarthritis | 715 | M15-M19 |
| skeletal | Rheumatoid arthritis | 714 | M05-M06 |
|  | Other Arthritis (Synovitis, Fibrositis, Connective tissue disorders, Ankylosing spondylitis, Gout Traumatic arthritis, pyogenic arthritis, Joint derangement, Dupuytren’s contracture, Other MSK disorders) | 727, 729, 710, 720, 274, 716, 711, 718, 728, 739 | M00-M03, M07, M10, M11-M14, M20-M25, M30-M36, M65-M79 |
|  | Osteoporosis | 733 | M81, M82 |

Adapted from previous studies (Kone-Penfoyo et al 2015/ Gruneir et al. 2016/ Mondor et al. 2017)

Individuals were assessed for a history of at least one of 18 chronic medical conditions. These conditions were selected based on their clinical relevance and burden, both in terms of cost and outcome (e.g. attributable deaths) as described in previous literature (Kone-Penfoyo et al 2015, Mondor et al 2017). We identified these chronic conditions using administrative data from Discharge Abstract Database (DAD) on April 1988 onwards and from Ontario Health Insurance Plan (OHIP) since July 1991 and onwards. For some of these chronic conditions, we used validated algorithms to ascertain cases and other conditions were ascertained according to inpatient hospital diagnostic codes (at least 1 from DAD) or outpatient physician billing codes (at least 2 from OHIP within a 2-year period). These conditions include arthritis (except rheumatoid arthritis), hypertension, Asthma, depression, diabetes, cancer, chronic coronary syndrome (CCS), cardiac arrhythmia, osteoporosis, chronic obstructive pulmonary disease (COPD), congestive heart failure (CHF), renal failure, dementia, mood and anxiety disorders, other mental health conditions, rheumatoid arthritis, stroke, acute myocardial infarction (AMI), Irritable Bowel Disease (IBD). The table below list the diagnostic codes to define the chronic conditions, and the clinical clusters in which each condition was included.

**Appendix 4. Supplementary tables**

*Table S1. Clinical characteristics of the cohort by linguistic group - (N=510,685)*

|  | | **Anglophone** *(N= 409,578)* | | | **Francophone** *(N= 11,907)* | | **Allophone** *(N=89,200)* | |  | **Total** *(N=510,685)* | |
| --- | --- | --- | --- | --- | --- | --- | --- | --- | --- | --- | --- |
|  | | *#* | *%* | | *#* | *%* | *#* | *%* |  | *#* | *%* |
| **Activities of daily living** | | |  |  | |  |  |  |  |  |  |
| Independent | | 254,701 | 62.2 | | 7,464 | 62.7 | 44,306 | 49.7 |  | *306,471* | *60.0* |
| Supervision required | | 45,745 | 11.2 | | 1,389 | 11.7 | 12,615 | 14.1 |  | *59,749* | *11.7* |
| Limited impairment | | 55,335 | 13.5 | | 1,635 | 13.7 | 16,191 | 18.2 |  | *73,161* | *14.3* |
| Extensive assistance | | 39,860 | 9.7 | | 1,090 | 9.2 | 11,748 | 13.2 |  | *52,698* | *10.3* |
| Dependent/total dependence | | 13,937 | 3.4 | | 329 | 2.8 | 4,340 | 4.9 |  | *18,606* | *3.6* |
| **Cognitive Performance Scale** | | |  |  | |  |  |  |  |  |  |
| Intact | | 184,726 | 45.1 | | 4,528 | 38.0 | 30,916 | 34.7 |  | *220,170* | *43.1* |
| Borderline intact | | 74,132 | 18.1 | | 2,237 | 18.8 | 17,299 | 19.4 |  | *93,668* | *18.3* |
| Mild impairment | | 114,996 | 28.1 | | 3,976 | 33.4 | 29,026 | 32.5 |  | *147,998* | *29.0* |
| Moderate/moderate severe impairment | | 26,245 | 6.4 | | 873 | 7.3 | 8,206 | 9.2 |  | *35,324* | *6.9* |
| Severe/very severe impairment | | 9,478 | 2.3 | | 293 | 2.5 | 3,753 | 4.2 |  | *13,524* | *2.6* |
| **CHESS score** | |  |  | |  |  |  |  |  |  |  |
| 0 (no instability) | | 83,935 | 20.5 | | 2,151 | 18.1 | 17,302 | 19.4 |  | 103,388 | 20.2 |
| 1 | 124,107 | 30.3 | | 3,171 | 26.6 | 28,264 | 31.7 |  | 155,542 | 30.5 |  |
| 2 | 111,583 | 27.2 | | 3,353 | 28.2 | 25,425 | 28.5 |  | 140,361 | 27.5 |  |
| 3 | | 68,602 | 16.8 | | 2,446 | 20.5 | 13,914 | 15.6 |  | 84,962 | 16.6 |
| 4 | | 19,426 | 4.7 | | 725 | 6.1 | 4,034 | 4.5 |  | 24,185 | 4.7 |
| 5 (highest level instability) | | 1,925 | 0.5 | | 61 | 0.5 | 261 | 0.3 |  | 2247 | 0.4 |
| **RUG-III** | |  |  | |  |  |  |  |  |  |  |
| Behavioural Problems | | 4,116 | 1.0 | | 163 | 1.4 | 1,139 | 1.3 |  | *5,418* | *1.1* |
| Clinically Complex | | 111,923 | 27.3 | | 3,337 | 28.0 | 16,463 | 18.5 |  | *131,723* | *25.8* |
| Extensive Care | | 4,258 | 1.0 | | 176 | 1.5 | 755 | 0.9 |  | *5,189* | *1.0* |
| Impaired Cognition | | 19,335 | 4.7 | | 657 | 5.5 | 6,324 | 7.1 |  | *26,316* | *5.2* |
| Reduced Physical Function | | 228,500 | 55.8 | | 6,778 | 56.9 | 56,620 | 63.5 |  | *291,898* | *57.2* |
| Rehabilitation | | 33,462 | 8.2 | | 645 | 5.4 | 6,289 | 7.1 |  | *40,396* | *7.9* |
| Special Care | | 7,980 | 2.0 | | 151 | 1.3 | 1,609 | 1.8 |  | *9,740* | *1.9* |
| **Prescribed drugs** | |  |  | |  |  |  |  |  |  |  |
| 0 | | 7,469 | 1.8 | | 138 | 1.2 | 1,495 | 1.7 |  | *9,102* | *1.8* |
| 1 | | 12,506 | 3.1 | | 228 | 1.9 | 2,632 | 3.0 |  | *15,366* | *3.0* |
| 2-3 | | 37,103 | 9.1 | | 685 | 5.8 | 8,245 | 9.2 |  | *46,033* | *9.0* |
| 4+ | | 352,499 | 86.1 | | 10,856 | 91.2 | 76,828 | 86.1 |  | *440,183* | *86.2* |

CHESS: Changes in Health, End-Stage Disease, Signs and Symptoms; RUG-III: Resource Utilization Groups version III (Ref. 19)

For some variables the numbers may not add up to 100% due to missing values

*Table S2. Top-five combinations of diseases across multimorbidity levels (2-5 diseases), by linguistic group (N=470,379)*

|  | **Anglophone** | | **Francophone** | | **Allophone** | |  |
| --- | --- | --- | --- | --- | --- | --- | --- |
|  | *#* | *%^*^* | *#* | *%^*^* | *#* | *%^*^* | *p-value^+^* |
| **Individuals with 2+ diseases** | **375,849** |  | **11,003** |  | **83,527** |  |  |
| Hypertension & Osteoarthritis | 9269 | 2.5 | 279 | 2.5 | 2331 | 2.8 | <.0001 |
| Cancer & Osteoarthritis | 4774 | 1.3 | 82 | 0.7 | 533 | 0.6 | <.0001 |
| Cancer & Hypertension | 3677 | 1.0 | 69 | 0.6 | 561 | 0.7 | <.0001 |
| Dementia & Hypertension | 1448 | 0.4 | 55 | 0.5 | 373 | 0.4 | <.0001 |
| Dementia & Osteoarthritis | 1162 | 0.3 | 48 | 0.4 | 269 | 0.3 | 0.0002 |
| **Individuals with 3+ diseases** | **325,253** |  | **9,636** |  | **74,011** |  |  |
| Hypertension & Osteoarthritis & Cancer | 6055 | 1.9 | 114 | 1.2 | 908 | 1.2 | <.0001 |
| Hypertension & Osteoarthritis & CHD | 3421 | 1.1 | 152 | 1.6 | 854 | 1.2 | <.0001 |
| Cancer & Osteoarthritis & Osteoporosis | 2478 | 0.8 | 71 | 0.7 | 1016 | 1.4 | <.0001 |
| Cancer & Osteoarthritis & Dementia | 2568 | 0.8 | 92 | 1.0 | 644 | 0.9 | 0.0005 |
| Hypertension & Osteoarthritis & Rheumatoid arthritis | 643 | 0.2 | 21 | 0.2 | 107 | 0.1 | 0.0241 |
| **Individual with 4+ diseases** | **254,230** |  | **7,650** |  | **58,565** |  |  |
| CHD & Diabetes & Hypertension & OA | 2638 | 1.0 | 110 | 1.4 | 929 | 1.6 | <.0001 |
| Cancer & Diabetes & Hypertension & OA | 2942 | 1.2 | 78 | 1.0 | 732 | 1.2 | 0.0006 |
| Diabetes & Dementia & Hypertension & OA | 1180 | 0.5 | 48 | 0.6 | 504 | 0.9 | <.0001 |
| CHD & Dementia & Hypertension & OA | 1026 | 0.4 | 25-30 | 0.3 | 240 | 0.4 | <.0001 |
| Osteoporosis & Dementia & Hypertension & OA | 773 | 0.3 | 20 | 0.3 | 247 | 0.4 | 0.0874 |
| **Individual with 5+ diseases** | **178,007** |  | **5,426** |  | **41,248** |  |  |
| Cancer & CHD & Diabetes & Hypertension & OA | 1391 | 0.8 | 33 | 0.6 | 304 | 0.7 | 0.298 |
| CHF & CHD & Diabetes & Hypertension & OA | 1111 | 0.6 | 47 | 0.9 | 395 | 1.0 | <.0001 |
| Arrhythmia & CHF & CHD & Hypertension & OA | 1210 | 0.7 | 32 | 0.6 | 311 | 0.8 | 0.2197 |
| CHD & Dementia & Diabetes & Hypertension & OA | 620 | 0.3 | 20-25 | 0.4 | 249 | 0.6 | <.0001 |
| Arrhythmia & CHD & Dementia & Hypertension & OA | 395 | 0.2 | 12 | 0.2 | 108 | 0.3 | 0.329 |

*^*^ Proportion from the total patients in the category in each stratum of linguistic group and multimorbidity status. Note that disease combination categories are not mutually exclusive within nor across level of multimorbidity.*

*OA: Osteoarthritis, CHF: congestive health failure, CHD: Coronary Heart Disease, COPD: chronic obstructive pulmonary disease.*

*^+^ Chi square test of homogeneity*

*Table S3. Risk of severe multimorbidity (5 or more chronic diseases) and linguistic characteristics (adjusted OR*, 95% CI)*

| **Patient characteristics** | **Odds Ratio** | **95%CI** | |
| --- | --- | --- | --- |
|  |  | **Lower** | **Upper** |
| Allophone vs. Anglophone | 1.04 | 1.02 | 1.06 |
| Francophone vs. Anglophone | 1.02 | 0.98 | 1.06 |
| **Age group** |  |  |  |
| 50-64 vs. <50 | 3.10 | 2.98 | 3.22 |
| 65+ vs. <50 | 6.96 | 6.71 | 7.22 |
| Female vs. Male | 0.80 | 0.79 | 0.81 |
| **Neighborhood income quintile** |  |  |  |
| Q1 vs. Q5 | 1.12 | 1.10 | 1.14 |
| Q2 vs. Q5 | 1.07 | 1.05 | 1.09 |
| Q3 vs. Q5 | 1.04 | 1.02 | 1.06 |
| Q4 vs. Q5 | 1.03 | 1.01 | 1.05 |
| Rural vs. Urban | 0.79 | 0.77 | 0.80 |
| Champlain vs. Southwest | 1.07 | 1.05 | 1.09 |
| North vs. Southwest | 0.91 | 0.89 | 0.93 |
| Immigrant vs. Canadian born | 0.34 | 0.32 | 0.35 |
| **Health characteristics** |  |  |  |
| CHESS 2 vs. No instability | 1.48 | 1.46 | 1.51 |
| CHESS 3 vs. No instability | 1.84 | 1.80 | 1.88 |
| CHESS 4 vs. No instability | 1.73 | 1.68 | 1.78 |
| CHESS 5 vs. No instability | 1.54 | 1.41 | 1.68 |
| CPS- Borderline intact vs. Intact | 1.14 | 1.13 | 1.16 |
| CPS-Mild impairment vs. Intact | 1.10 | 1.08 | 1.11 |
| CPS- Moderate impairm. vs. Intact | 1.10 | 1.07 | 1.13 |
| CPS- Mod. severe impairm. vs. Intact | 0.87 | 0.82 | 0.93 |
| CPS- Severe impairm. vs. Intact | 0.83 | 0.79 | 0.87 |
| CPS-Very severe impairm. vs. Intact | 0.90 | 0.81 | 1.01 |
| ADL-Supervision vs. Independent | 0.98 | 0.97 | 1.00 |
| ADL-Limited vs. Independent | 1.00 | 0.98 | 1.01 |
| ADL-Extensive 1 vs. Independent | 0.91 | 0.88 | 0.93 |
| ADL-Extensive 2 vs. Independent | 0.97 | 0.94 | 1.00 |
| ADL- Dependent vs. Independent | 0.99 | 0.95 | 1.03 |
| ADL-Total depend. vs. Independent | 0.94 | 0.85 | 1.03 |

** Multivariable logistic regression model, adjusted by age, sex, neighborhood income level, immigrant status, rurality, area, and health characteristics.*
